# Play interventions to reduce anxiety and negative emotions in hospitalized children

**DOI:** 10.1186/s12887-016-0570-5

**Published:** 2016-03-11

**Authors:** William H. C. Li, Joyce Oi Kwan Chung, Ka Yan Ho, Blondi Ming Chau Kwok

**Affiliations:** School of Nursing, The University of Hong Kong, 4/F, William M. W. Mong Block, 21 Sassoon Road, Pokfulam, Hong Kong, China; Playright Children’s Play Association (Playright), Hong Kong, China

**Keywords:** Anxiety, Children, Emotions, Hospitalization, Paediatrics, Play interventions

## Abstract

**Background:**

Hospitalization is a stressful and threatening experience, which can be emotionally devastating to children. Hospital play interventions have been widely used to prepare children for invasive medical procedures and hospitalization. Nevertheless, there is an imperative need for rigorous empirical scrutiny of the effectiveness of hospital play interventions, in particular, using play activities to ease the psychological burden of hospitalized children. This study tested the effectiveness of play interventions to reduce anxiety and negative emotions in hospitalized children.

**Methods:**

A non-equivalent control group pre-test and post-test, between subjects design was conducted in the two largest acute-care public hospitals in Hong Kong. A total of 304 Chinese children (ages 3-12) admitted for treatments in these two hospitals were invited to participate in the study. Of the 304 paediatric patients, 154 received hospital play interventions and 150 received usual care.

**Results:**

Children who received the hospital play interventions exhibited fewer negative emotions and experienced lower levels of anxiety than those children who received usual care.

**Conclusion:**

This study addressed a gap in the literature by providing empirical evidence to support the effectiveness of play interventions in reducing anxiety and negative emotions in hospitalized children. Findings from this study emphasize the significance of incorporating hospital play interventions to provide holistic and quality care to ease the psychological burden of hospitalized children.

**Trial registration:**

ClinicalTrials.gov NCT02665403. Registered 22 January 2016.

## Background

Hospitalization can be a threatening and stressful experience for children [1]. Because of unfamiliar with the environment and medical procedures and unaware of the reasons for hospitalization, it can result in children’s anger, uncertainty, anxiety, and feelings of helplessness [2, 3]. Anxiety is the most commonly reported of these negative responses, and high levels of anxiety can be harmful to children’s physiological and psychological health. Excessive anxiety also impedes children’s efficacy in coping with medical treatment, and increases their uncooperative behaviour and negative emotions towards healthcare professionals [1, 3, 4].

Play has long been regarded as a vital element in the normal growth and development of children [5, 6], and is widely used in many Western countries to alleviate the stress experienced by paediatric patients and their families during hospitalization [7]. Through play, children are given the opportunity to develop mastery of self and the environment and to enhance their understanding of the world [8].

Florence Nightingale, the founder of modern nursing, emphasized the essential nature of play for hospitalized children [1]. She also pointed out that it is the responsibility of healthcare professionals to create and maintain a therapeutic environment for paediatric patients [9]. Florence Erikson [10] was one of the first nurses to conduct a study of play interventions for hospitalized children. In exploring the reactions of children to the hospital experience, she showed that they found it easier to express their feelings about hospital when they were given the opportunity to play with clinical equipment [10]. Additionally, she demonstrated the benefits of using play interviews and dolls to prepare hospitalized children for invasive medical procedures [10].

Wolfer and Visintainer [11] conducted an influential study to examine the stress responses and adjustment to hospitalization of paediatric surgical patients. The results showed that children who received psychological preparation, including hospital play interventions, in contrast to those who did not, reported fewer upset behaviour and post-hospital adjustment problems, but more cooperation with the hospital. Since then, a number of studies on hospital play interventions have been carried out preparing children for invasive medical procedures and helping them cope with the stress of hospitalization [1, 12, 13]. Nonetheless, a review of the literature reveals that the majority of such studies were case studies [3], and there is little scientific evidence to find out the precise clinical effectiveness of hospital play [14].

Zahr [13] conducted a study on preparing pre-school children to undergo surgery by means of hospital play interventions. The researcher found that children who received such interventions pre-operatively experienced fewer adverse behavioural changes and were significantly calmer post-operatively than children who received only routine care. One limitation of this study was that only pre-school children were involved, the benefits of hospital play for school-age children remaining uncertain. Li and his colleagues conducted the first randomized controlled trial to test the effects of hospital play interventions on children undergoing day surgery [3]. The researchers found that these children experienced less anxiety and exhibited fewer negative emotions than children receiving only information preparation in the pre- and post-operative periods. Nevertheless, the generalizability of this study was limited because only minor elective surgery was involved and the children did not stay in hospital overnight. Another similar randomized controlled trial was conducted by He and colleagues to test the effectiveness of hospital play interventions on the outcomes of children undergoing in-patient elective surgery [12]. The results were consistent with Li’s study, showing that patients who received the interventions had fewer negative emotions and experienced less anxiety than those receiving routine nursing care. Nevertheless, both studies [3, 12] were focused on preparing children for surgery, and the effects of hospital play interventions in helping children cope with the general stress of hospitalization remain uncertain. There is an imperative need for rigorous quantitative research into the efficacy of hospital play interventions in hospitalized children.

It is widely thought that Chinese people are influenced by Confucianism, which emphasizes balance and harmony achieved through the concepts of ‘chung’ and ‘yung’ in everyday life [15–17]. In this way, disease is regarded as arising from ‘bad spirits’, and that exercise will only aggravate it and break the rules of harmony [17]. Under this cultural influence, Hong Kong Chinese parents or even healthcare professionals are accustomed to advise hospitalized children to take more rest and not to engage in any energy-consuming activities, such as playing. Traditionally, most Chinese parents and some healthcare professionals view play as less important than medical treatment or physiological care [18]. It is unclear therefore, whether it is appropriate or feasible to incorporate play as a psychological intervention for hospitalized children into the Hong Kong Chinese context. The aim of this study was to test the effectiveness of hospital play interventions in minimizing the anxiety levels and negative emotions of hospitalized Hong Kong Chinese children.

### Hypotheses of the intervention

The two hypotheses were:

1. Children who received the hospital play interventions would exhibit fewer negative emotions when compared with children who received usual care.
2. Children who received the hospital play interventions would experience lower levels of anxiety when compared with children who received usual care.

#### Theoretical framework

The theory of cognitive appraisal, stress and coping [19] was used to guide the study. This transactional model is frequently applied to research on children [12, 18]. According to Lazarus and Folkman, cognitive factors are primarily responsible for determining the impact of stress and the experience of stress is dependent on individuals’ interpretation of a potentially threatening event and their psychological, behavioural and emotional responses to it.

It is well known that hospitalization is highly stressful for children and can have adverse effects on their health [1, 3]. Lazarus and Folkman claim that individuals’ evaluation of a potential threat is influenced by their perceptions of control over that threat. Previous studies have indicated that a lack of control over the hospital setting and upcoming medical procedures is a major source of stress, which may cause substantial anxiety for hospitalized children [18]. There is evidence that individuals with adequate self-control over a potential threat would encourage a positive coping strategy, consequently improve their psychological health [20]. The goal of the hospital play interventions in this study was therefore to help children maintain control through familiarization and rehearsal over the hospital setting and medical procedures. We also believed that through participating in hospital play activities children would enhance their interpersonal skills and social and creative abilities. Children not only had fun, but were also encouraged to desensitize stressful situations and to instil greater self-control over the new situation that they were involved in. The interventions gave the children an opportunity to practice medical or nursing routines through play and allowed them to interact actively with the environment in a non-threatening way.

## Methods

### Design

A quasi-experimental study was conducted in two public hospitals in different areas of Hong Kong, with one assigned as the ‘control’ and the other as the ‘experimental’ hospital. The two hospitals have similar paediatric specialties, settings, and medical and nursing care.

### Participants

The participants were recruited from these two public hospitals, and were eligible if they were Chinese children aged between 3 and 12, able to speak Cantonese, and required to stay in hospital for at least three consecutive days. We excluded those children with identified cognitive and learning difficulties.

To ensure sufficient power to detect differences between the groups, power analysis was used to estimate the sample size. With reference to the previous study that had examined the effects of hospital play interventions on children’s anxiety levels and emotions when undergoing day surgery [3], we predicted a medium effect size for the differences between the two groups. To predict this effect size at a 5 % significance level (*p* < 0.05) and power of 0.80, we calculated that 64 subjects would be required in each group [21]. We allowed for a potential attrition rate of 15 % and thus 11 additional participants per group were needed, giving a total sample size of 150. However, according to Piaget [6], children aged 3-7 and 8-12 are at the preoperational and concrete operational stages of development, respectively. Because children’s cognitive development is linked to their age, different methods of assessment were used for the two age groups. Data analyses were therefore performed separately for the two age groups, 3-7 and 8-12. For these reasons, we aimed to recruit at least 300 subjects (150 in each age group) to this study.

### Intervention

In the control group, children received standard medical and nursing care, such as vital signs observation, pharmacological treatment and wound and pain management.

In the experimental group, participants received hospital play interventions, conducted by hospital play specialists. To ensure that the play intervention dosage – in terms of the frequency and duration– would adequately assess outcomes such as children’s anxiety levels and emotions, a meeting was set up between the Playright and research team. The Playright is a professional organization that organizes a variety of children’s play programs for the public, and offers education and training to different professionals and organizations in Hong Kong. Taking into consideration the busy clinical setting and the adequate dosage of play interventions, we proposed each participant to receive continuously 30 min of hospital play interventions each day. Such interventions (sometimes referred to as ‘therapeutic play interventions’) are activities designed for preparing children psychologically for hospitalization according to their levels of psychosocial and cognitive development and to health-related issues [7]. The interventions in this study consisted of structured and non-structured activities. Most hospitalized children received play interventions together in a playroom, except those required to stay in bed would be given play interventions at bedside. Parents were encouraged to stay with their children during play interventions. Although the intervention protocol was standardized for different medical procedures, play specialists would select appropriate play activities based on the age, diagnosis and physical condition of the children, who were also given a choice of activities. For example, play specialists would engage younger children in play, such as puppets and toy blocks, to obtain more sensory experiences. For older children, play specialists would offer them activities with high cognitive demand, such as word and board games. The specialists also logged the timing, duration, and nature of play for each child. Examples of the interventions are described in Table 1.

**Table 1:** Examples of the Hospital Play Interventions

Type of Play	Objectives	Activities	
Preparation Play	• To increase children’s understanding of medical procedures • To give children a sense of control over threatening events and help to clarify their misconceptions	Go through every step of a medical procedure using different instruments, such as tailor-made pretend medical dolls, procedural orientation books, real medical equipment, and miniature medical equipment	
Medical Play	• To facilitate children’s expression of their concerns and feelings related to hospitalization • To familiarize children with the hospital environment and routine medical procedures • To facilitate children’s expression of their feelings and emotions related to hospitalization	•Provide various real and/or toy medical equipment (e.g. stethoscope, syringe without needles, bandages, medical cup, gloves, mask, nurse’s cap, dressing pack, etc.) during children’s hospitalization • Get children involved in different kinds of expressive play activities (e.g. painting, singing, dancing, journaling, sand play, puppets, etc.), and encourage them to share or express their feelings	
Distraction Play	• To reduce the anxiety of children undergoing medical procedures	Provide interesting games and toys (e.g. blowing bubbles, pop-up books, puppets, computer games, music, video, sensory toys, relaxation techniques, etc.) to distract children’s attention from medical procedures	
Developmental Play	• To promote optimum psychosocial development and prevent regression among hospitalized children	Involve children in appropriate play activities (e.g. toys, board games, story books, arts and crafts play, etc.) according to their ages and abilities	

* Photos adopted with permission from Playright – Taken from Hospital Play Service pamphlet

### Measures

#### Visual Analogue Scale (VAS)

Anxiety levels of children aged between 3 and 7 were assessed by using the VAS, which consists of a 10 cm horizontal line on a piece of card, with different facial expressions supplemented by the words ‘I have no anxiety’ at one end and ‘I have so much anxiety’ at the other. Higher scores represent higher levels of anxiety. Children aged between 5 and 7 used a movable indicator to indicate their levels of anxiety. However, as children aged 3-4 could be confused by the scale because of their limited verbal expression and cognitive capacities, their anxiety levels were assessed and rated by their parents, using the VAS. The VAS is a widely used assessment tool as it is not complicated to administer, easy for children to understand, and is a valid method for assessing subjective feelings [22, 23]. One advantage of using the VAS is that it is unaffected by the limited test-taking skill of young children. Previous studies have shown the VAS to be a valid, reliable, and sensitive tool for assessing individual subjective feelings [22–24].

#### Chinese version of the State Anxiety Scale for Children (CSAS-C)

Anxiety levels of children aged between 8 and 12 were assessed by using the short-form Chinese version of the State Anxiety Scale for Children (CSAS-C). This consists of 10 items scored from 1 to 3, with total scores ranging from 10 to 30. Higher scores represent greater anxiety [25]. The short form of the CSAS-C has undergone psychometric testing [26] and results showed satisfactory internal consistency reliability (*r* = .83). Adequate concurrent validity was confirmed by comparing the short with the full form (*r* = .92). Construct validity was confirmed by means of confirmatory factor analysis [27].

#### Children’s Emotional Manifestation Scale (CEMS)

The emotions of the hospitalized children were assessed using the CEMS, which is an observation scale. The CEMS consists of five categories, each category scored from 1 to 5, with summed scores from 5 to 25. Higher scores represent more negative emotional behaviour. The CEMS has undergone psychometric tests [28] and results show satisfactory internal consistency reliability (*r* = .92) and adequate inter-rater reliability (*r* = .96). Construct validity was confirmed by comparing the state anxiety scores with the CEMS (*r* = .76).

### Process evaluation

To identify the strengths and weaknesses of the hospital play interventions from the participants’ perspective, a process evaluation was conducted with five children aged between 8 and 12 and with the parents of five children aged between 3 and 12 in the experimental group. They were randomly selected to attend a short one-to-one semi-structured interview according to the serial codes generated by a computer. The process evaluation could help to optimize the quality of the interventions, and to determine whether its delivery is feasible and acceptable to participants. All of the interviews were conducted by a full-time research assistant, who had substantial knowledge and experience in conducting interviews. The interviews were taped-recorded.

### Data collection

A research assistant collected demographic data from the parents and from the children’s medical records after obtaining the consent form. The children’s baseline anxiety levels were also documented. For the experimental group, the interventions started after the baseline data had been collected. The emotional behaviour of each child was observed by a research assistant for two consecutive days, at the end of which a research assistant documented the child’s overall emotional behaviour, using the CEMS. The child’s anxiety levels were reassessed and documented. Semi-structured interviews were conducted with selected children and their parents.

### Data analysis

The Statistical Package for Social Science (SPSS) software, IBM version 20.0 for Windows was used for the data analysis. Descriptive statistics were used to calculate the means, standard deviations, and ranges of the scores on the various scales. The homogeneity of the two groups was examined using inferential statistics (independent *t*-test and χ^2^). The interrelationships among the scores on the different scales and the demographic variables were assessed using the Pearson product-moment correlation coefficient. Differences in the mean scores on the CEMS and the children’s anxiety levels between the two intervention groups were investigated by an independent *t*-test and mixed between-within subjects ANOVA, respectively. Multiple regression analysis was performed to examine the effects of participants’ demographic and clinical characteristics on the outcome measures.

The following measures were taken to reduce potential bias during the data collection. First, the research assistant responsible for data collection received training from the researcher, in particular concerning the method of assessment. Second, the research assistant was requested to follow the guidelines strictly in every assessment. Additionally, the researcher periodically checked the correctness of the assessment method used by the research assistant.

## Ethical approval

To conduct this study in the two public hospitals, ethical approval from the Hospital Authority, West Cluster Research Committee (KWC-REC) was sought. Reference: KW/FR-12-020 (55-06). Date of approval: 7th December 2012. To ensure the rights of all participants were protected, especially for the vulnerable subjects such as children in this study, the researchers strictly adhered to the Declaration of Helsinki (http://www.wma.net/en/30publications/10policies/b3/index.html.pdf?print-media-type&footer-right=[page]/[toPage]) and the ethical principles in designing and conducting clinical research. We informed parents of the purpose of the study and then obtained their written consent. Children were also invited to put their names on a special individual assent form told that their participation was voluntary. Both parents and their children were told that they had the right to withdraw from the study at any time and were assured of the confidentiality of their data. In addition, parents of all the children in the images plus all the adults in the images (Table 1) gave written informed consent for the images to be used or published.

## Results

A total of 393 patients were recruited from November 2012 to October 2013. However, 89 questionnaires were incomplete as a result of unexpected early discharge or intra-hospital transfer. Only 304 questionnaires were thus retained for analysis. Of the 304 patients, 154 received the interventions and 150 received standard care. The demographic and baseline anxiety scores for the experimental and control groups for both 3 to 7 and 8 to 12 year-old age groups are shown in Table 2. The results show that the experimental and control groups in the two age groups were similar in respect of the children’s ages, sex, diagnoses, number of hospital admissions, and baseline anxiety scores, indicating a high level of comparability of variance between the two groups.

**Table 2:** Demographic and baseline characteristics between the experimental and control groups for the two age groups of 3 to 7 years and 8 to 12 years

	*n* (%)		*χ* ^2^	*p*	*n* (%)		*χ* ^2^	*p*
	Ages 3-7				Ages 8-12			
	Experimental (*n* = 103)	Control (*n* = 79)			Experimental (*n* = 51)	Control (*n* = 71)		
Gender								
Male	58 (56.0)	44 (56.0)	0.02	0.89 ^*ns*^	29 (56.9)	43 (60.5)	0.14	0.61 ^ns^
Female	45 (44.0)	35 (44.0)			22 (43.1)	28 (39.5)		
Diagnosis								
Respiratory problem	37 (35.9)	28 (35.5)	0.08	0.99 ^*ns*^	21 (41.1)	28 (39.4)	0.15	0.97 ^ns^
Gastroenterology problem	23 (22.3)	17 (21.5)			14 (27.5)	19 (26.8)		
Genitourinary problem	13 (12.6)	11 (13.9)			8 (15.7)	13 (18.3)		
Household accident	12 (11.7)	9 (11.4)			3 (5.9)	4 (5.6)		
Fever for investigation	18 (17.5)	14 (17.7)			5 (9.8)	7 (9.9)		
Number of hospital admissions								
1	57 (55.3)	39 (49.4)	0.05	0.86 ^*ns*^	27 (52.9)	38 (53.5)	0.03	0.74 ^ns^
2-3	31 (30.1)	27 (34.2)			14 (27.5)	24 (33.8)		
4-5	10 (9.7)	9 (11.4)			7 (13.7)	5 (7.1)		
6 or above	5 (4.9)	4 (5.0)			3 (5.9)	4 (5.6)		
	*M* (*SD*)		*t*	*p*	*M* (*SD*)		*t*	*p*
Age	4.7 (1.4)	4.5 (1.5)	−0.98	0.33 ^*ns*^	9.8 (1.3)	10.9 (2.1)	−0.55	0.59 ^*ns*^
Mean anxiety scores	6.7 (2.4)	6.9 (2.5)	0.75	0.46 ^*ns*^	22.5 (4.3)	23.1 (4.5)	0.30	0.77 ^*ns*^

^*Notes: ns*^ *=* Not significant at *p* > 0.05

The relationships among the scores on the different scales and the demographic variables were examined. Correlation coefficients of .10 to .29, .30 to .49, and .50 to 1.0 were referred to as small, medium, and large effects, respectively (Cohen, 1992). The results showed that there were statistically significant high positive correlations between the anxiety and CEMS scores of children aged 3-7 (*r* = .62, *n* = 182, *p* = .01) and 8-12 (*r* = .70, *n* = 122, *p* = .01). Small negative correlations were found between the time of hospital admission and anxiety scores for children aged 3-7 (*r* = −0.26, *n* = 182, *p* < .01) and 8-12 (*r* −0.28, *n* = 122, *p* < .01). The results of the multiple regression analyses indicated that demographic and clinical factors, including the children’s age and gender, diagnosis and time of hospital admission, did not make a statistically significant contribution to the prediction of anxiety and CEMS scores.

The mean anxiety and emotional manifestation scores of children aged 3-7 and 8-12 in the experimental and control groups are shown in Table 3. An independent *t*-test showed statistically significant differences between the mean CEMS scores of children aged 3-7 in both groups [*t* (180) = −7.3, *p* < .001], and of children aged 8-12 in both groups [*t* (120) = −8.1, *p* < .001]. Children receiving the interventions exhibited less negative emotional behaviour during hospitalization. A mixed between-within-subjects ANOVA was performed on the anxiety scores. The results (Table 4) showed that hospitalized children (both 3-7 and 8-12 age groups) who participated in the interventions experienced significantly lower levels of anxiety than those receiving standard care only. With reference to the guidelines proposed by Cohen [29], the eta squared indicates a moderate effect size for the interventions on the children’s levels of anxiety in both age groups.

**Table 3:** The Means and Standard Deviations for the Anxiety Scores in Children Across Two Time Periods and Emotional Manifestation Scores between the Experimental and Control Groups

	*Mean, SD*			
	Experimental		Control	
	Ages 3-7 (*n* = 103)	Ages 8-12 (*n* = 79)	Ages 3-7 (*n* = 51)	Ages 8-12 (*n* = 71)
Anxiety scores:				
Baseline	6.7, 2.4	22.5, 4.3	6.9, 2.5	23.1, 4.5
Post-interventions	3.9, 1.7	19.3, 3.8	6.3, 2.4	22.7, 4.3
Emotional Manifestation scores	9.4, 1.9	10.8, 2.7	12.6, 3.4	13.7, 3.8

**Table 4:** The results of mixed between-within-subjects ANOVA on the scores for anxiety levels in children ages 3–7 and 8-12

	Ages 3-7			Ages 8-12		
	*F*-value	*P*-value	*Eta squared*	*F*-value	*P*-value	*Eta squared*
Main effect for time	63.3	.005	0.12	50.8	.008	0.11
Main interaction effect	1.1	.009	0.16	23.7	.006	0.18
Main effect for intervention	78.7	.03	0.06	6.4	.02	0.07

Effect size (eta squared) conventions: small effect = 0.01; moderate effect = 0.06; large effect = 0.14

### Process evaluation

#### Perception of the play interventions by parents and their children

When the children were asked to comment on the hospital play interventions in the hospital, many of them stated that it helped them to know more about medical procedures. With such an understanding, the children said they no longer felt anxious and stressed before their medical procedures. An example of a child’s response is given below.

*“I was not going to worry about venipuncture after the hospital play interventions. The play specialist explained the procedures to me during the interventions. I don’t get scared of syringes anymore. They can be fun, like a toy. I also made a syringe doll by myself with the help of the play specialist.”*

Similar thoughts were shared by the parents. Many of them stated that their children became much more settled after the interventions and some said that their children were much more courageous about having medical procedures. Some examples of their responses are given below.

*“My son became much more settled after the hospital play interventions. He felt much happier after playing with the play specialist.”*

*“I’ve never seen my son so brave when facing medical procedures. I would like to thank the play specialist for her hospital play interventions.”*

Both the children and their parents reported that their impressions of the hospital changed after the interventions. Before the interventions, many of them perceived that healthcare professionals, particularly doctors and nurses, were apathetic and not sensitive enough to patients’ psychological needs. As such, they did not feel able to ask questions during the medical procedure or the consultation. After the interventions, most of them had changed their mind and said that they felt the hospital did care about their psychological needs. An example of a child’s response is given below.

*“I am not afraid to see doctors now. I am able to express my feelings and ask them questions. I was startled by my last experience in another hospital because they (healthcare professionals) didn’t care what I thought. However, I wasn’t stressed this time because they (the play specialists) have been helping me to cope with the pain (caused by medical procedures).”*

#### Suitability of the hospital play interventions

Many of the children and their parents said they were happy to receive the hospital play interventions because the activities were fun and interesting. Some examples of their responses are given below.

*“I like having the hospital play interventions – it’s fun. I no longer feel bored and lonely after such an intervention.”*

*“It (the hospital play interventions) is a wonderful service for my child; I am satisfied with the activities and games because they are fun and interesting.”*

Some parents mentioned that the hospital play interventions provided an opportunity for their children to socialize with others. During the interventions, they could make friends with other children on the ward so that they felt less lonely. An example of their responses is given below.

*“Since the hospital play interventions, my daughter does not mind having to stay longer in the hospital. She made friends with the girl who was next to her and the play specialist during the interventions.”*

## Discussion

The findings, in accord with previous studies, suggest that the Hong Kong Chinese children experienced considerable high anxiety on admission to hospital [3, 18]. Compared with previous studies using the same scale to assess the anxiety of Hong Kong Chinese children of a similar age, the anxiety scores of children on admission to hospital in this study were similar to those of children immediately before undergoing surgery, but higher than those of school children before an academic assessment [3]. The results also showed a high positive correlation between the CSAS-C and CEMS. The findings concur with those of previous studies [3, 12], in which children with high anxiety levels had more negative emotional responses. The findings provide further evidence that anxiety impedes children’s ability to cope with hospitalization and medical procedures, and increases negative emotions toward healthcare.

There is an assumption that the number of hospital admissions might strongly affect children’s anxiety levels and emotional responses. Nevertheless, the results of the multiple regression analysis did not find any effects from the number of hospital admissions on children’s outcomes. The findings suggest that hospitalization is a stressful experience for children whether they have had previous hospital admission experience or not.

The overall findings support the effects of hospital play interventions in minimizing the anxiety levels and negative emotions of Hong Kong Chinese children who have been hospitalized. Indeed, providing play for hospitalized children has special advantages as illness, stress and physical restriction hinder their accustomed play and socialization, which are crucial for the normal growth and development of children. Most importantly, involvement in play activities while in hospital can enhance children’s coping skills and relieve their stress, leading to improved psychosocial adjustment both to their illness and to the fact of hospitalization.

### Implementation potential of hospital play in clinical settings

Although the overall results support these interventions in reducing children’s anxiety and negative emotions, it is essential to assure the implementation potential of such interventions in clinical settings. Allowing flexible time for the interventions with repeated sessions is crucial to assure hospitalized children are able to engage in play activities. Children in this study were only invited to join the play activities when they were not occupied in any medical and nursing procedure. It would be practical, therefore, for healthcare professionals to consider incorporating hospital play as a routine psychological preparation for children in their care.

Most of the children interviewed reported that the hospital play interventions helped to relieve their anxiety because they gained more knowledge about their illness and familiarized themselves with the medical procedures. Most children were pleased to know that most of the medical or nursing procedures would not cause them pain, or that any pain that might occur would be well controlled. Most importantly, the children enjoyed play in the hospital and found the activities fun and interesting.

Although Chinese parents have traditionally overlooked the importance of play for hospitalized children, most parents in the experimental group appreciated the availability of play activities within the hospital. A previous local study showed that parents were reassured as they saw their child participate in plays activities and interacted with other children [3].

Some healthcare professionals may have concerns that extra manpower is required and more support from the hospital administration is needed if play is implemented within the hospital. However, using hospital play specialists (HPS) may be one of the best solutions to the shortage of manpower. Indeed, most hospitals in Australia, the United States, and other Western countries employ HPS, who play an important role in promoting psychological care for hospitalized children through the provision of play activities. Although it may require some extra resources in the short term, it would certainly enhance quality health delivery. Indeed, it only takes 30 min a day to implement play activities to make the life of a hospitalized child less difficult. Certainty, it would be economically feasible for health organizations to consider and implement play interventions as standard practice for hospitalized children.

#### Limitations

There are some limitations to this study. First, although a randomized controlled trial is the most sophisticated method of testing causal relationships between independent and outcome variables, to randomize individual patients within a hospital paediatric unit is not feasible as there is a chance of contamination between different treatment groups in the same setting, and because some parents might be confused if they realize their child is receiving a different form of interventions. A quasi-experimental design was therefore used. Second, the use of convenience sampling and play interventions was only implemented in one hospital, which limits the ability of the study to generalize its results**.** Third, the study only observed children’s anxiety levels and emotional responses on two consecutive days, which may not have been long enough to assess fully the effect of hospitalization on their psychological well-being. Nevertheless, as short-stay hospitalization is recommended in today’s healthcare policy, many children will be discharged home after one to three days.

#### Practical implications

Play is instinctive, voluntary, and spontaneous; children play just as birds fly and fish swim [30]. The findings of the study generate new knowledge and evidence about hospital play, with major clinical implications. We believe that hospital play interventions can be applied to all children, regardless of different cultural backgrounds or settings. Given the importance of play to children’s psychological health, it is recommended that the Hospital Authority in Hong Kong should recognize this importance by providing more resources and establish more space and facilities for children to play when they are in hospital. Most importantly, it is crucial to employ HPS to facilitate the integration of play into routine care for hospitalized children.

## Conclusions

Despite some possible limitations, this study has bridged a gap in the literature by examining the effects of hospital play interventions on the outcomes of hospitalized children. The results emphasize the significance of incorporating hospital play to provide holistic and quality care to ease the psychological burden of hospitalized children. It also promotes the knowledge and understanding among both healthcare professionals and parents that play is of paramount importance to children’s lives, and that they need to play even when they are sick.

## References

[1] Francischinelli AGB, Almeida FA, Fernandes DMSO (2012). Routine use of therapeutic play in the care of hospitalized children: nurses’ perceptions. Acta Paulista de Enfermagem.

[2] Fernandes SC, Arriaga P (2010). The effects of clown intervention on worries and emotional responses in children undergoing surgery. J Health Psychol.

[3] Li HCW, Lopez V, Lee TLI (2007). Effects of preoperative therapeutic play on outcomes of school-age children undergoing day surgery. Res Nurs Health.

[4] Lohaus A, Klein-Hessling J, Ball J, Wild M (2004). The prediction of health-related in behavior in elementary school. J Health Psychol.

[5] Erikson E (1963). Childhood and society.

[6] Piaget J (1963). The origins of intelligence in children.

[7] LeVieux-Anglin L, Sawyer EH (1993). Incorporating play interventions into nursing care. Pediatr Nurs.

[8] Norris AE, Aroian KJ, Warren S, Wirth J (2012). Interactive performance and focus groups with adolescents: The power of play. Res Nurs Health.

[9] Frauman A, Gilman C (1989). Creating a therapeutic environment in a pediatric renal unit. ANNA J.

[10] Erickson F (1958). Reactions of children to hospital experience. Nurs Outlook.

[11] Wolfer JA, Visintainer MA (1975). Pediatric surgical patients’ and parents’ stress responses and adjustments. Nurs Res.

[12] He HG, Zhu L, Li HCW, Wang W, Vehviläinen-Julkunen K, Chan SW (2014). A randomized controlled trial of the effectiveness of a therapeutic play intervention on outcomes of children undergoing inpatient elective surgery: study protocol. J Adv Nurs.

[13] Zahr LK (1998). Therapeutic play for hospitalized preschoolers in Lebanon. Pediatr Nurs.

[14] Carroll J (2000). Evaluation of therapeutic play: a challenge for research. Child Fam Soc Work.

[15] Chan EA, Cheung K, Mok E, Cheung S, Tong E (2006). A narrative inquiry into the Hong Kong Chinese adults’ concepts of health through their cultural stories. Int J Nurs Stud.

[16] Li WHC, Chien WT (2009). The importance of incorporating cultural issues into nursing interventions for Chinese populations. Strategies in evaluation of complex health care interventions for people with physical or mental health issues.

[17] Nisbett R (2003). The Geography of Thought: How Asians and Westerners Think Different--and Why.

[18] Li HCW, Chung OKJ, Ho KY, Chiu YS (2011). Effectiveness and Feasibility of using the Computerized Interactive Virtual Space in Reducing Depressive Symptoms of Hong Kong Chinese Children Hospitalized with Cancer. J Spec Pediatr Nurs.

[19] Lazarus RS, Folkman S (1984). Stress, appraisal, and coping.

[20] Dempster M, McCorry NK, Brennan E, Donnelly M, Murray LJ, Johnston BT (2011). Do changes in illness perceptions predict changes in psychological distress among oesophageal cancer survivors?. J Health Psychol.

[21] Polit DF, Beck CT (2012). Nursing research: Principles and methods.

[22] Lara-Muñoz C, Ponce De Leon S, Feinstein AR, Puente A, Wells CK (2004). Comparison of three rating scales for measuring subjective phenomena in clinical research. I. use of experimentally controlled auditory stimuli. Arch Med Res.

[23] Li HCW, Mak YW, Chan SCS, Chu AK, Lee EY, Lam TH (2013). Effectiveness of a play-integrated primary one preparatory programme to enhance a smooth transition for children. J Health Psychol.

[24] Davey HM, Barratt AL, Butow PN, Deeks JJ (2007). A one-item question with a Likert or Visual Analog Scale adequately measured current anxiety. J Clin Epidemiol.

[25] Li HCW, Chung OKJ, Ho KY (2013). Effectiveness of an adventure-based training programme in promoting the psychological well-being of primary schoolchildren. J Health Psychol.

[26] Li HCW, Lopez V (2007). Development and validation of a short form of the Chinese version of the State Anxiety Scale for Children. Int J Nurs Stud.

[27] Li HCW, Wong EML, Lopez V (2008). Factorial structure of the Chinese version of the State Anxiety Scale for Children (short form). J Clin Nurs.

[28] Li HCW, Lopez V (2005). Children’s Emotional Manifestation Scale: Development and testing. J Clin Nurs.

[29] Cohen J (1992). A power primer. Psychol Bull.

[30] Landreth GL (2012). Play therapy [electronic resource]: the art of the relationship.

